# Robust Fusion of Diffusion MRI Data for Template Construction

**DOI:** 10.1038/s41598-017-13247-w

**Published:** 2017-10-11

**Authors:** Zhanlong Yang, Geng Chen, Dinggang Shen, Pew-Thian Yap

**Affiliations:** 1 School of Marine Science and Technology, Northwestern Polytechnical University, Xi’an, 710072 China; 2 Department of Radiology and Biomedical Research Imaging Center (BRIC), University of North Carolina at Chapel Hill, NC, 27599 USA; 3 Department of Brain and Cognitive Engineering, Korea University, Seoul, 02841 Korea

## Abstract

Construction of brain templates is generally carried out using a two-step procedure involving registering a population of images to a common space and then fusing the aligned images to form a template. In practice, image registration is not perfect and simple averaging of the images will blur structures and cause artifacts. In diffusion MRI, this is further complicated by intra-voxel inter-subject differences in fiber orientation, fiber configuration, anisotropy, and diffusivity. In this paper, we propose a method to improve the construction of diffusion MRI templates in light of inter-subject differences. Our method involves a novel *q*-space (i.e., wavevector space) patch matching mechanism that is incorporated in a mean shift algorithm to seek the most probable signal at each point in *q*-space. Our method relies on the fact that the mean shift algorithm is a *mode seeking* algorithm that converges to the mode of a distribution and is hence robust to outliers. Our method is therefore in effect seeking the most probable signal profile at each voxel given a distribution of signal profiles. Experimental results show that our method yields diffusion MRI templates with cleaner fiber orientations and less artifacts caused by inter-subject differences in fiber orientation.

## Introduction

Brain templates^1,2^ capture the common features of a population of images and play crucial roles in the processing and analysis of brain images. They are widely used for guiding brain tissue segmentation, normalization of images to a common space, and brain labeling with regions of interest. Unlike templates of anatomical T1-weighted images, diffusion MRI templates afford additional white matter microstructural information that can be harnessed for tissue characterization and axonal tracing. To ensure that the microstructural information captured at each voxel location is properly encoded in the template, dedicated techniques are needed.

**Figure 1:**
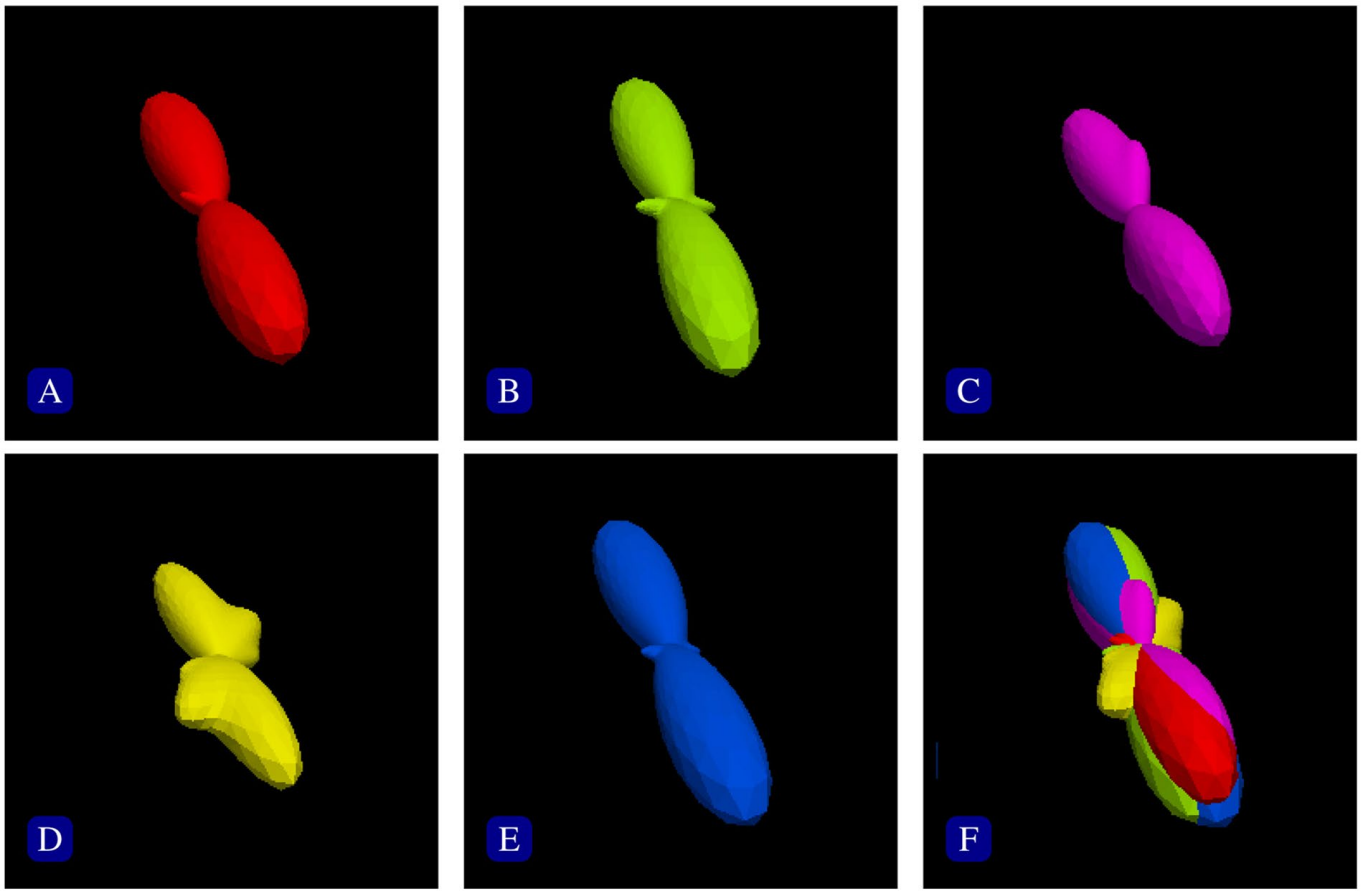
Inter-subject variation. The orientation distribution functions (ODFs) from a single voxel of the spatially registered diffusion MRI datasets of five subjects (**A**–**E**), overlapped in (**F**). The shapes and orientations of the ODFs vary across subjects.

Template construction generally involves fusing a population of images that are aligned to a common space. The major challenge is to retain the fine anatomical details during the template construction process, which is often affected by inaccurate image registration, especially in highly convoluted cortical regions. Many methods have been proposed to improve the quality of the constructed template^3–8^. They sought accurate image alignment to create templates with sharp anatomical details. To this end, nonlinear image registration^9–13^ is often adopted for better alignment accuracy. However, in practice, perfect registration is difficult, if not impossible. Averaging misaligned images to construct a template blurs details and introduces artifacts. In diffusion MRI^14^, the problem is even more challenging, since the alignment of gross anatomical structures does not necessarily guarantee the alignment of the microstructural information captured in each voxel. For example, Fig. 1 shows that the orientation distribution functions (ODFs) at a common voxel location might differ in orientation and configuration across subjects. In this situation, it is unclear for example how signals characterizing fiber bundles of varying orientations, which can occur naturally across subjects, should be fused to form the template. Moreover, the commonly used simple averaging method is sensitive to outliers. For instance, if the distribution of signal profiles of single-directional fiber bundles is contaminated with a small number of signal profiles of crossing fibers, simple averaging will result in a crossing profile, albeit with a small secondary peak. This outcome apparently is not representative of the majority.

In this paper, we propose a novel *q*-space patch-matching mechanism that is incorporated in a mean shift algorithm to seek the most probable signal at each point in *q*-space. Mean shift is a versatile non-parametric iterative algorithm that can be used for mode seeking^15^. Instead of the mean, our method employs the mean shift algorithm to determine the mode of a distribution of signal profiles. The mean shift algorithm uses a kernel to measure the distance between signals. To increase robustness to noise, we measure the distance between signals using patches defined in the *q*-space. Patch matching is key to the success of many state-of-the-art denoising algorithms, such as non-local means^16,17^. Patch matching in *q*-space is performed with the help of azimuthal equidistant projection^18^ and rotation invariant features^19^. Experimental results focusing on inter-subject differences in fiber orientation confirm that our method yields diffusion templates with cleaner fiber orientations and are less susceptible to artifacts caused by inter-subject differences. A preliminary version of this work has been presented in a workshop^20^. Herein, we provide a more comprehensive evaluation using more sophisticated synthetic data and high-quality data from the Human Connectome Project (HCP)^21^. The relevant experimental results, analyses, and discussions are new and not part of our workshop publication.

## Results

Quantitative and qualitative experiments using synthetic and real data were performed to evaluate the proposed template construction method. For quantitative evaluation, we use the peak signal-to-noise ratio (PSNR, in dB) as the metric:

1 $${\rm{PSNR}}=10\cdot {\mathrm{log}}_{10}\,(\frac{{{\rm{MAX}}}^{2}}{{\rm{MSE}}}),$$

where MSE is the mean squared error and MAX is the maximum possible signal value. The MSE is computed as the average squared difference calculated across voxels and gradient directions.

### Synthetic Data Experiment

**Figure 2:**
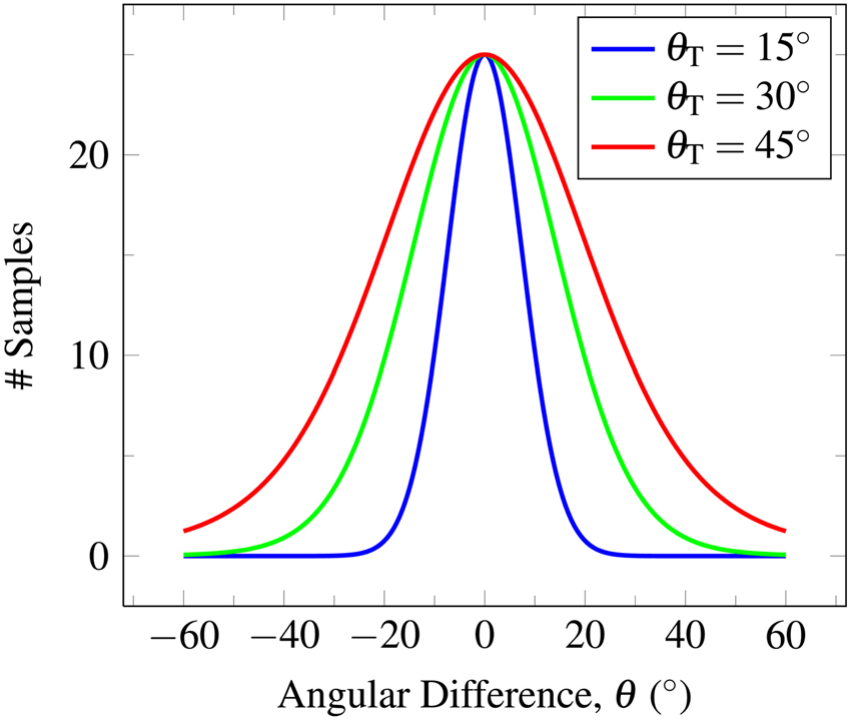
The distribution of Watson probability distribution function. The distribution of orientations according to the Watson probability distribution function with different values for parameter *θ*_T_.

**Figure 3:**
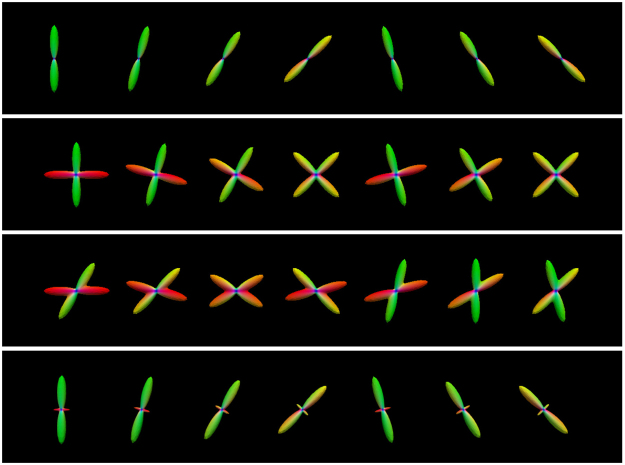
Synthetic dataset. Examples from the synthetic dataset simulating (first row) one direction, (second row) two equally-weighted directions 90° apart, (third row) two equally-weighted directions 60° apart, and (fourth row) two unequally-weighted directions 90° apart.

The dataset was simulated with *b* = 3,000 s/mm^2^ and 81 non-collinear gradient directions. In order to simulate the dispersion of fiber orientations across subjects, we generated a set of diffusion signal profiles of fiber bundles oriented according to the Watson probability distribution function^22^, which in modified form is given as

2 $$f(\theta |\kappa )\propto \exp \,[-\kappa (1-{\cos }^{2}\,(\theta ))],$$

where *θ* is the angle of deviation from the ground truth direction and the concentration parameter *κ* is defined as $\kappa =\mathrm{2(1}-{\cos }^{2}\,({\theta }_{{\rm{T}}}{))}^{-1}$. Parameter *θ*_T_ determines the degree of dispersion of the orientations of the fiber bundles. The distributions for *θ*_T_ = 15°, 30°, 45° are shown in Fig. 2. Based on the resulting fiber orientations, we use a multi-tensor model^23^ to generate the diffusion signal profiles. The axial and radial diffusivities are estimated from the corpus callosum of the real data, which is described in the next section. The *b*-value is chosen to match the real data. The fiber ODFs^24,25^ of diffusion profiles with single direction, two equally weighted directions (60° and 90° apart), and two unequally weighted directions (90° apart) are shown in Fig. 3. The “template” is computed using this distribution of diffusion signal profiles and the outcome is compared with the ground truth without deviation. Four levels of Rician noise (3%, 5%, 7% and 9%) were added to the noise free dataset. Rician noise was simulated by adding Gaussian noise (i.e. ${\mathscr{N}}\mathrm{(0},v(p\mathrm{/100))}$) to the complex domain of the signal with noise variance determined by noise-level percentage *p* and maximum signal value *v* (150 in our case).

**Figure 4:**
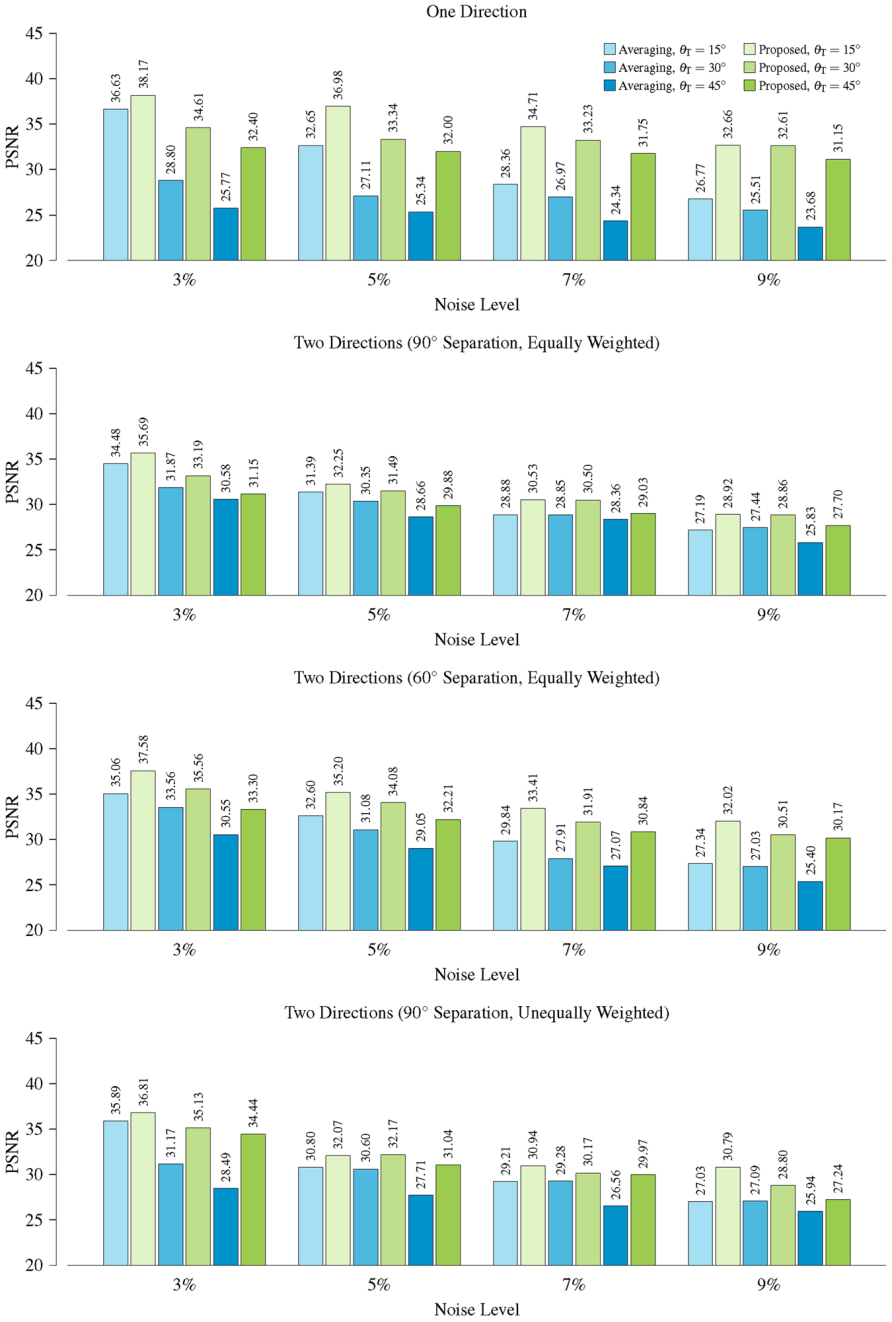
PSNR comparison. PSNR comparison of results given by simple averaging and the proposed method using the synthetic data.

**Figure 5:**
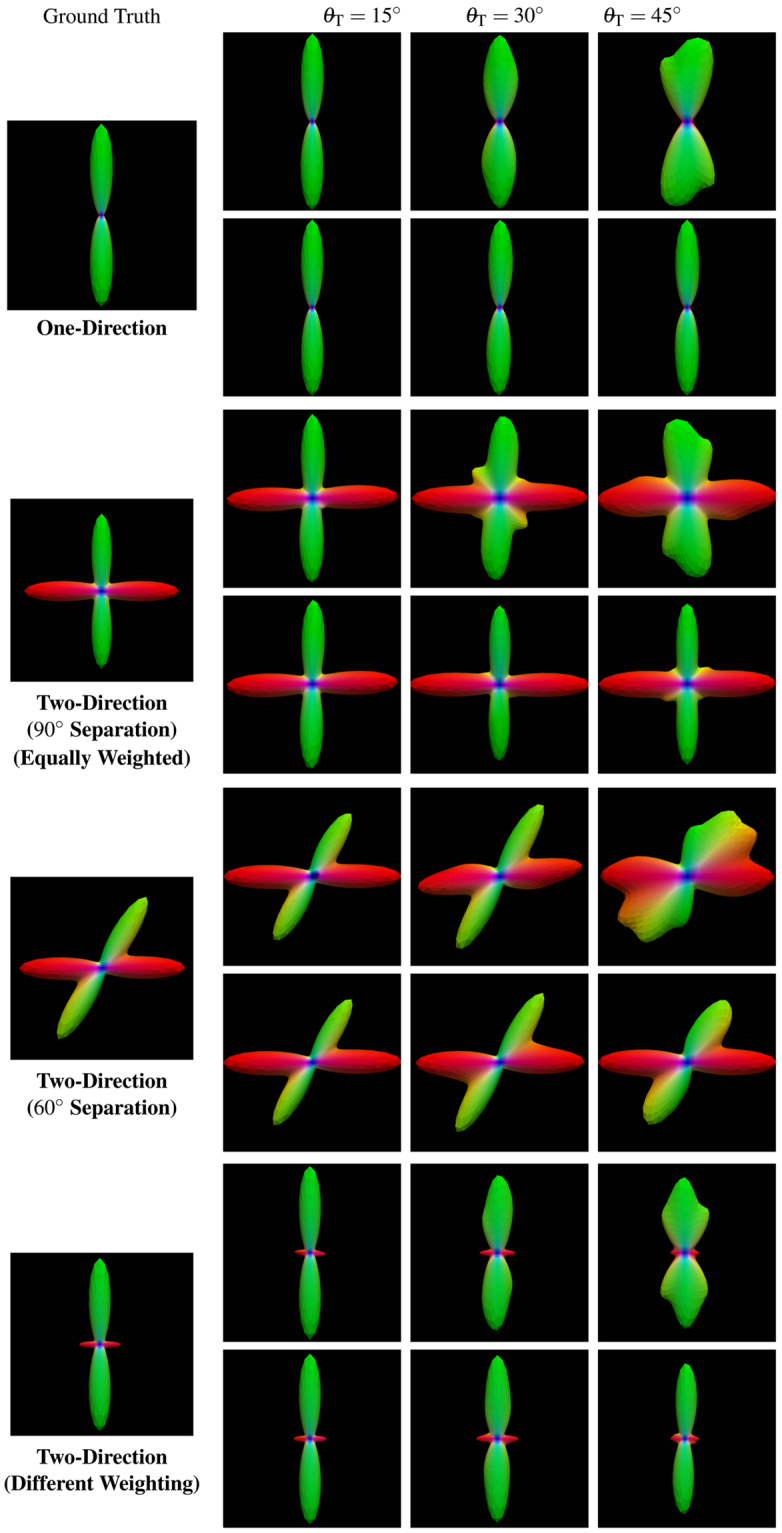
ODF comparison. For each case, the ground truth is shown on the far left for reference, the top row shows the results for simple averaging and the bottom row shows the results for the proposed method. The results were generated using the synthetic dataset with 5% noise and *θ*_T_ = 15°, 30°, 45°.

As shown in Fig. 4, for the various noise levels, our method improves the PSNRs over simple averaging for both one- and two-directional cases. The PSNR improvement is over 2 dB and sometimes even up to 7 dB. The fiber ODFs of some representative results, shown in Fig. 5, indicate that simple averaging causes artifacts and that the proposed method yields results that are very close to the ground truth.

We further show in Table 1 the orientational discrepancy (OD)^10^ between the fiber orientation estimates^24,25^ computed from the signal profiles generated using the two methods and the ground truth. Suppose that ${{\mathscr{G}}}_{1}$ and ${{\mathscr{G}}}_{2}$ are the sets of directions obtained from the local minima of two ODFs, OD^10^ is defined as

3 $${\rm{OD}}=\frac{1}{2}\,[\mathop{{\rm{\max }}}\limits_{{{\bf{g}}}_{1}\in {{\mathscr{G}}}_{1}}\,\mathop{{\rm{\min }}}\limits_{{{\bf{g}}}_{2}\in {{\mathscr{G}}}_{2}}\,d({{\bf{g}}}_{1},{{\bf{g}}}_{2})+\mathop{{\rm{\max }}}\limits_{{{\bf{g}}}_{2}\in {{\mathscr{G}}}_{2}}\,\mathop{{\rm{\min }}}\limits_{{{\bf{g}}}_{1}\in {{\mathscr{G}}}_{1}}\,d({{\bf{g}}}_{2},{{\bf{g}}}_{1})],$$

where $d({{\bf{g}}}_{1},{{\bf{g}}}_{2})={\cos }^{-1}\,(|{{\bf{g}}}_{1}\cdot {{\bf{g}}}_{2}|)$ is the angle difference between **g**_1_ and **g**_2_.

**Table 1:** The average OD between fiber orientation estimates and the ground truth.

*θ*_T_	Noise	Single Direction		90° Separation (Equally Weighted)		60° Separation (Equally Weighted)		90° Separation (Unequally Weighted)	
		Averaging (°)	Proposed (°)	Averaging (°)	Proposed (°)	Averaging (°)	Proposed (°)	Averaging (°)	Proposed (°)
15°	3%	0.0455	**0**.**0182**	0.1353	**0**.**0348**	0.0380	**0**.**0371**	0.0337	**0**.**0227**
	5%	0.0599	**0**.**0267**	0.1496	**0**.**0437**	0.2351	**0**.**1643**	0.1052	**0**.**0410**
	7%	0.0658	**0**.**0458**	0.2107	**0**.**1814**	0.3802	**0**.**1766**	0.3129	**0**.**2341**
	9%	0.2506	**0**.**1157**	0.2780	**0**.**1830**	2.9184	**0**.**2912**	0.3737	**0**.**2381**
30°	3%	0.0696	**0**.**0381**	0.0701	**0**.**0635**	2.7727	**0**.**1057**	0.1180	**0**.**0652**
	5%	0.1677	**0**.**0804**	0.2521	**0**.**1863**	3.1273	**0**.**1856**	0.2342	**0**.**0756**
	7%	3.8070	**0**.**2173**	3.9694	**0**.**2314**	3.9297	**1**.**9592**	3.7208	**0**.**1542**
	9%	7.0452	**0**.**2596**	6.6786	**2**.**4269**	8.2865	**3**.**8886**	4.0710	**0**.**2392**
45°	3%	0.0765	**0**.**0554**	2.8817	**0**.**0931**	6.7836	**0**.**0797**	0.0751	**0**.**0317**
	5%	3.8793	**0**.**0831**	3.8226	**0**.**2400**	25.7778	**3**.**1191**	0.2293	**0**.**0792**
	7%	7.1966	**0**.**0968**	7.2697	**2**.**8693**	27.5585	**4**.**0779**	7.2373	**0**.**1717**
	9%	11.0748	**0**.**3150**	15.1399	**5**.**4962**	28.7225	**6**.**0309**	11.0473	**0**.**2234**

As Table 1 shown, the two methods give reasonably good results at low level of noise and when the dispersion is small. However, when the noise level and dispersion increase, the OD values given by simple averaging are significantly higher than the proposed method.

### Real Data Experiment

Further evaluation was performed using the diffusion-weighted (DW) images of 20 subjects from the HCP^21^. The 1.25 × 1.25 × 1.25 mm^3^ data were acquired with diffusion weightings *b* = 1000, 2000, 3000 s/mm^2^, each in 90 non-collinear gradient directions. 18 baseline images with low diffusion weighting *b* = 5 s/mm^2^ were also acquired. To reduce memory cost and computational burden, we only use the *b* = 3,000 s/mm^2^ shell in our evaluation. Prior to template construction, the DW images were registered to the FSL fractional anisotropy (FA) standard space (http://fsl.fmrib.ox.ac.uk/fsl/fslwiki/FMRIB58_FA) via diffeomorphic demons^12^ using the FA images. Based on the estimated deformation fields, the DW images were warped to the standard space and reorientated^26^.

**Figure 6:**
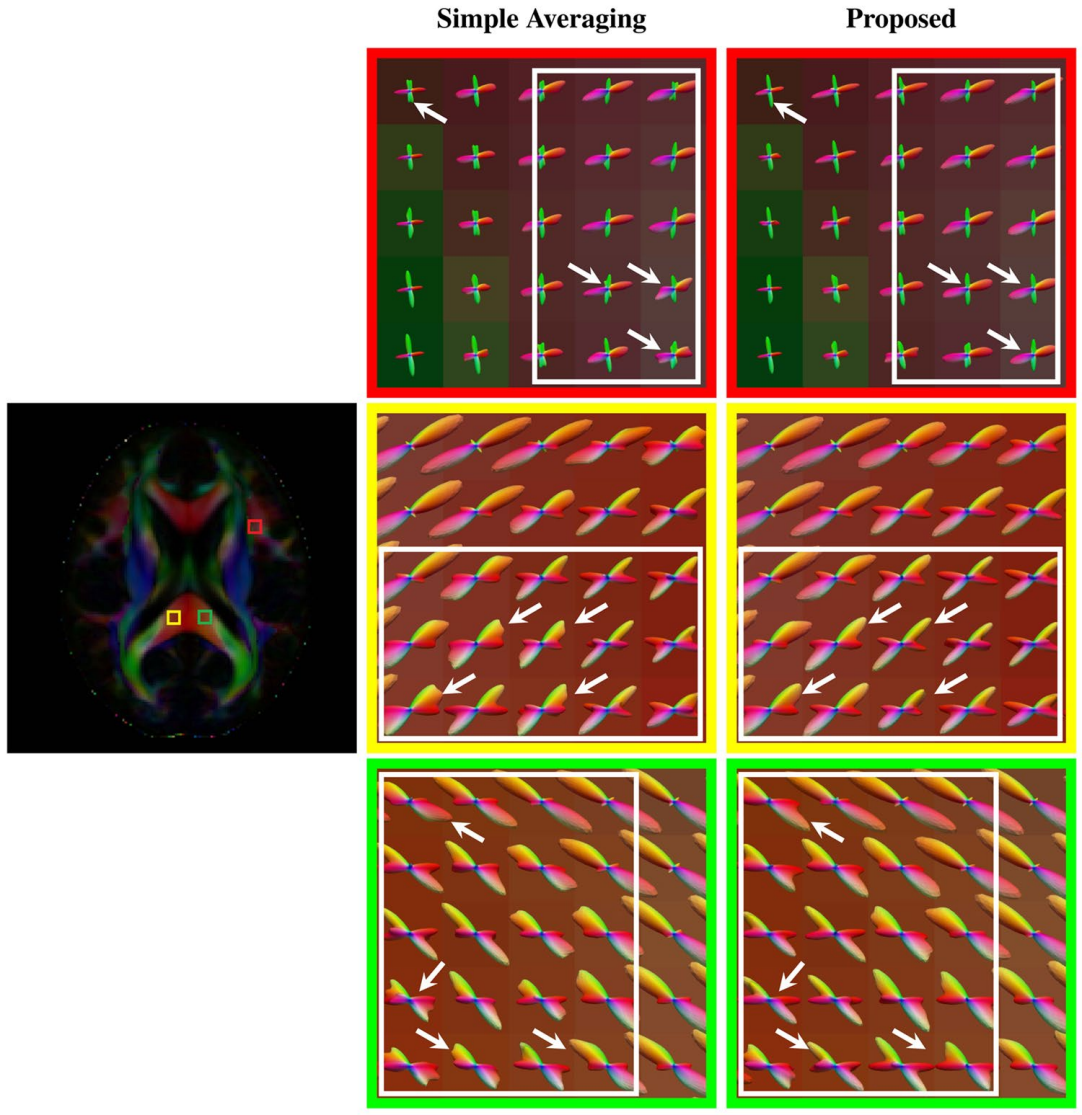
Axial view of ODFs. ODFs estimated using simple averaging and the proposed method. Visible improvements are marked by arrows.

**Figure 7:**
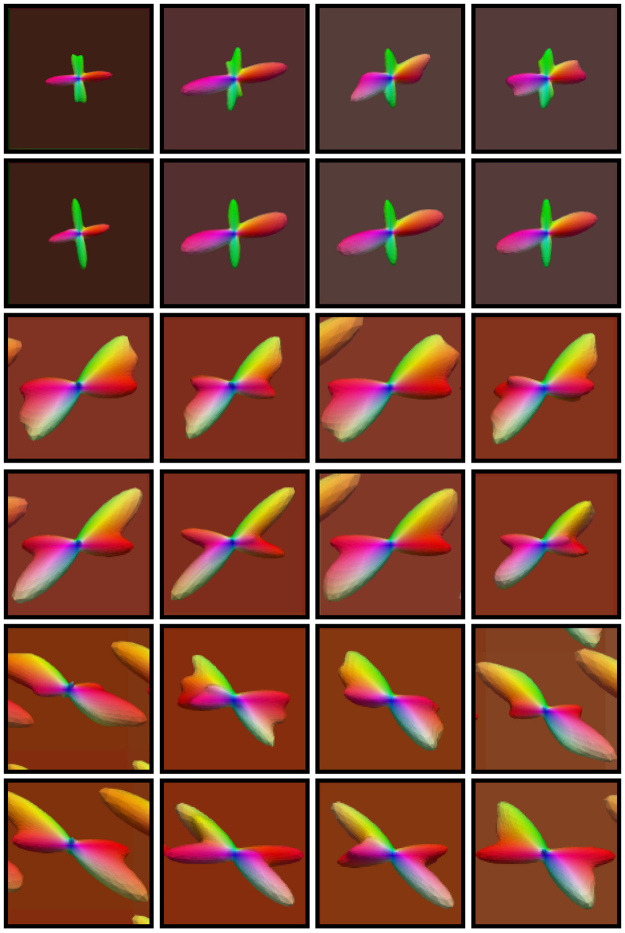
Axial view of ODFs (close-ups). Rows 1, 3, and 5 are the results given by simple averaging and rows 2, 4, and 6 by the proposed method.

**Figure 8:**
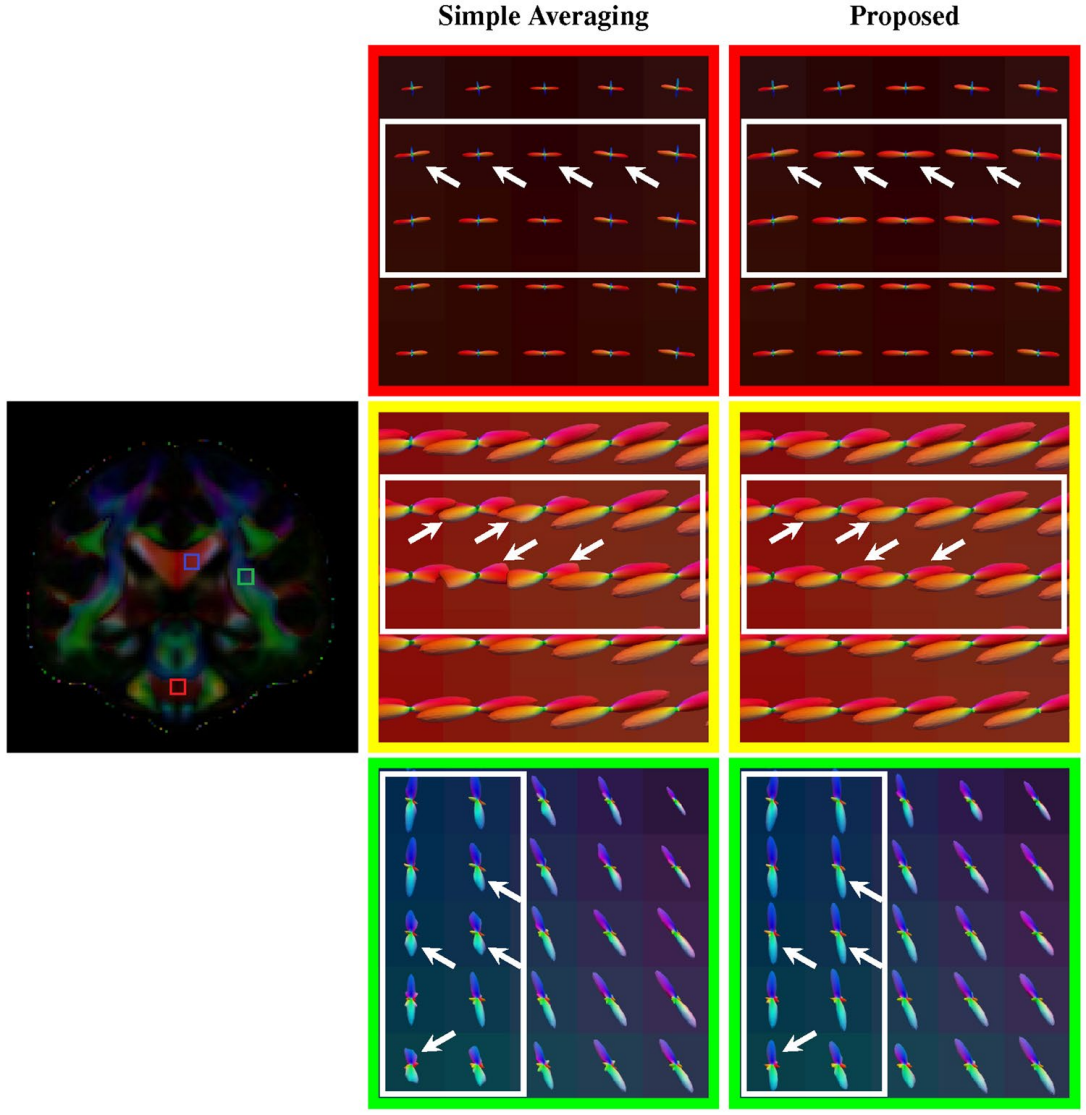
Coronal view of ODFs. Similar to Fig. 6, but in coronal view. Visible improvements are marked by arrows.

**Figure 9:**
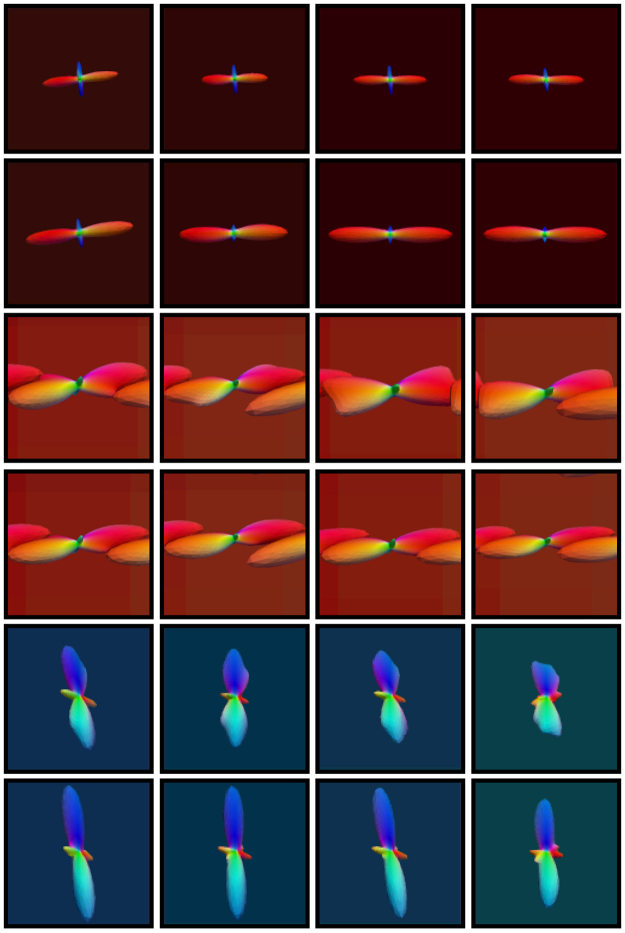
Coronal view of ODFs (close-ups). Rows 1, 3, and 5 are the results given by simple averaging and rows 2, 4, and 6 by the proposed method.

**Figure 10:**
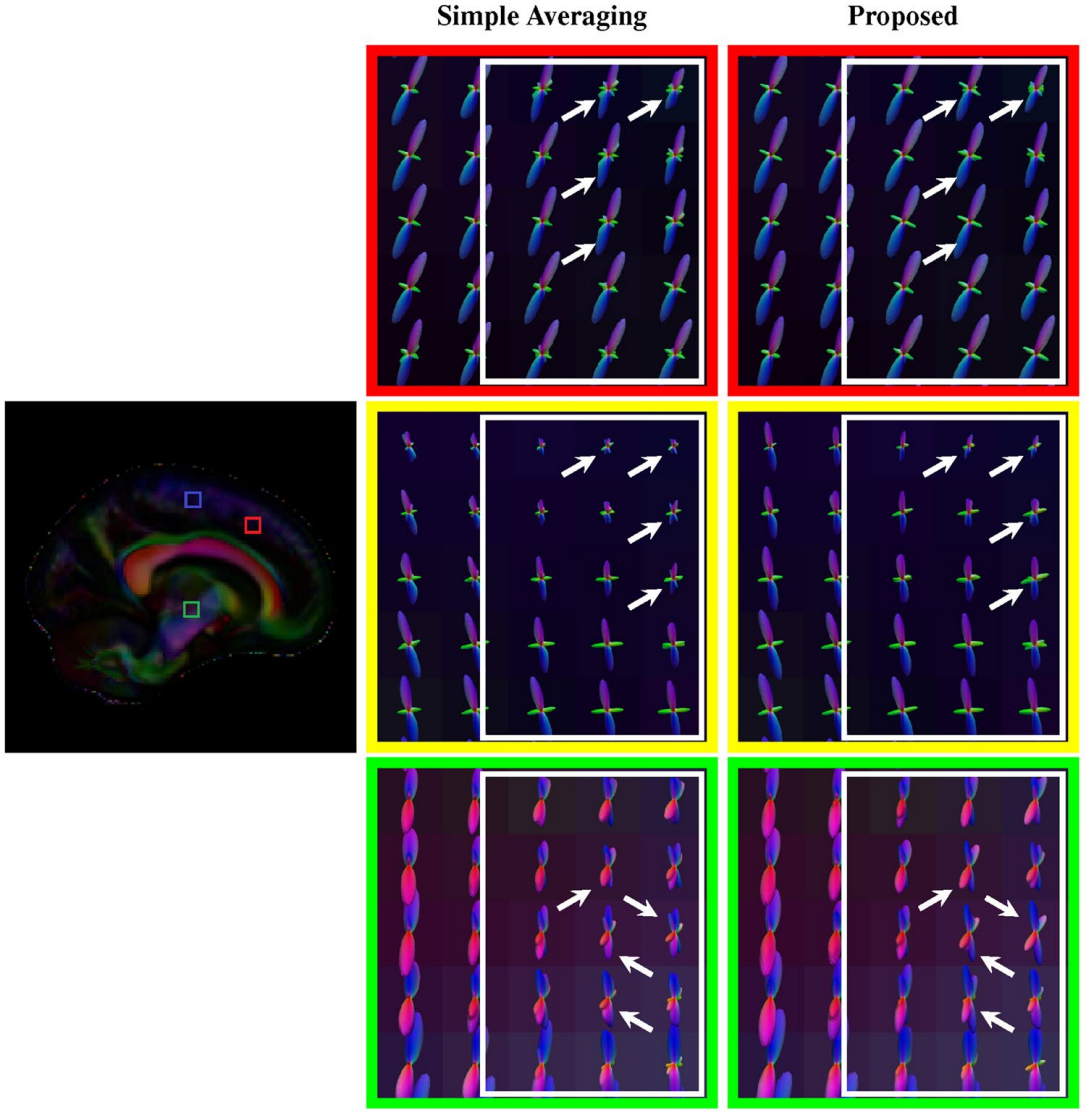
Sagittal view of ODFs. Similar to Fig. 6, but in sagittal view. Visible improvements are marked by arrows.

**Figure 11:**
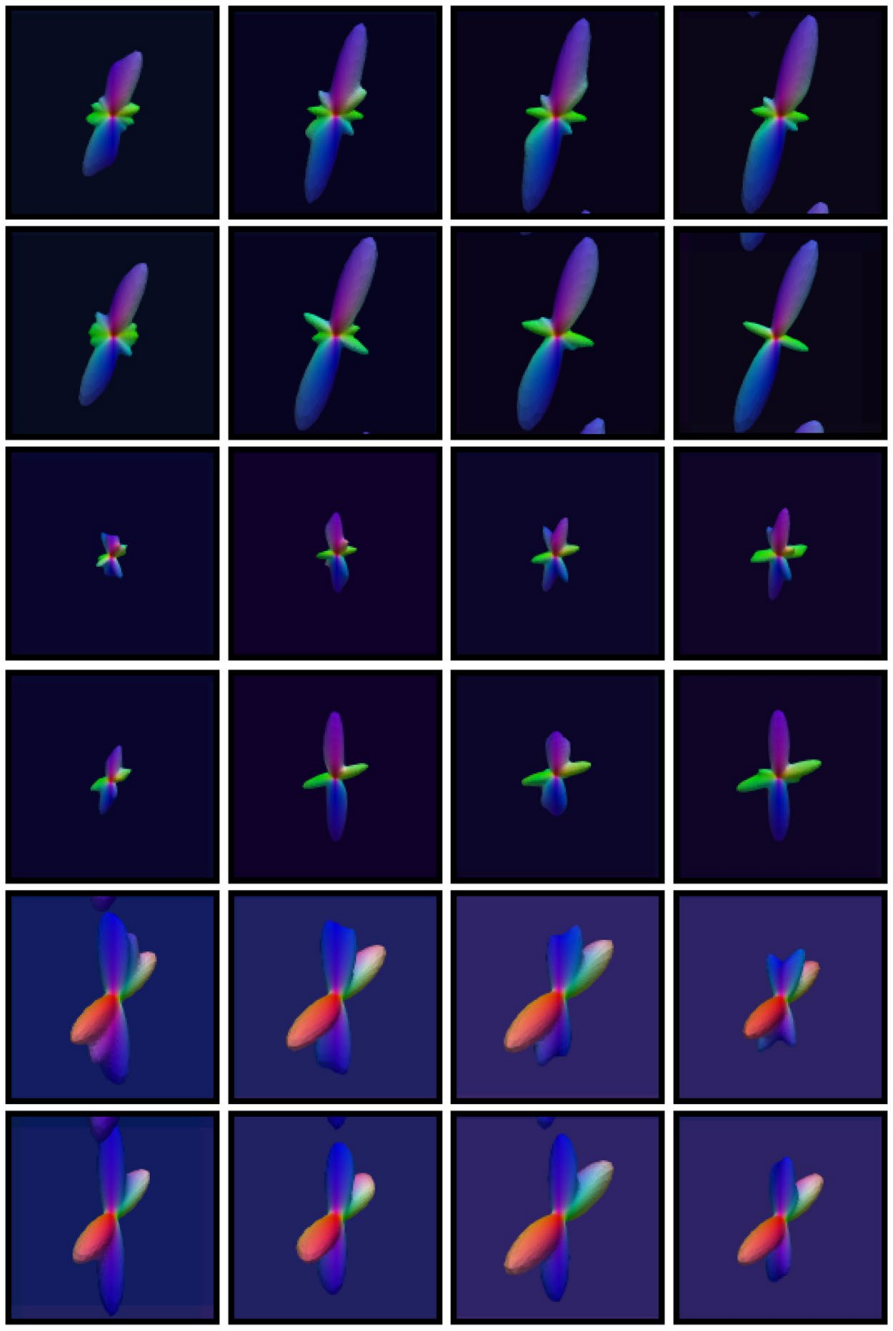
Sagittal view of ODFs (close-ups). Rows 1, 3, and 5 are the results given by simple averaging and rows 2, 4, and 6 by the proposed method.

As shown in Fig. 6 (axial, close-ups in Fig. 7), Fig. 8 (coronal, close-ups in Fig. 9) and Fig. 10 (sagittal, close-ups in Fig. 11), our method produces ODFs with more consistent directions and less spurious peaks. Visible differences are marked using arrows and boxes. For simple averaging, the ODF glyphs are generally shorter, indicating less directionality. On the other hand, our method gives sharper and longer glyphs. Simple averaging is found to cause spurious peaks, whereas the proposed method shows clean results with clear directions. See, for example, the ODFs marked by arrows in the green region of interest (ROI) shown in Fig. 10.

**Figure 12:**
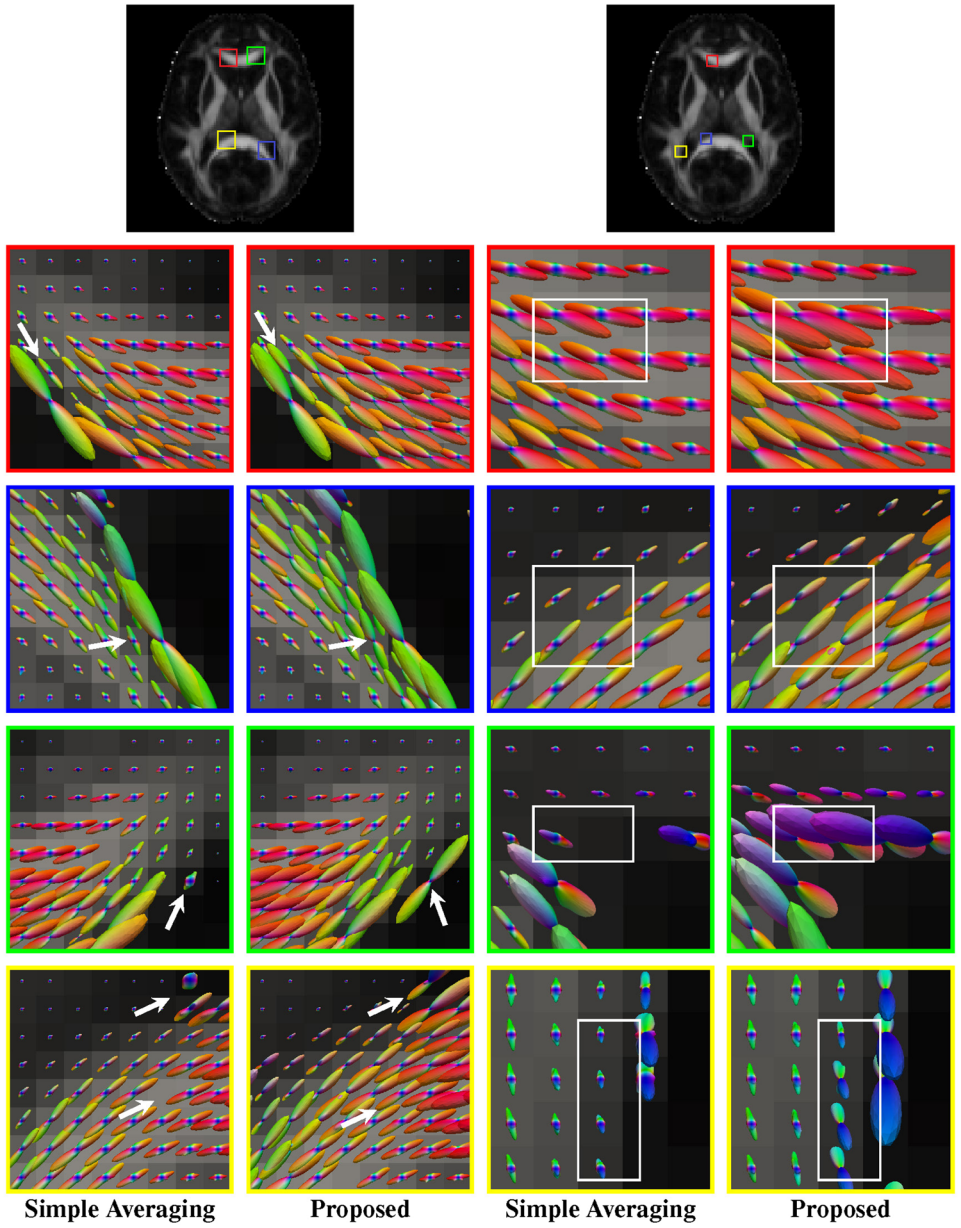
Normal-quality data. Comparisons of white matter fiber ODFs given by the simple averaging method (columns 1 and 3) and our method (columns 2 and 4). The fractional anisotropy images at the top are shown for reference. Visible differences between the methods are marked by arrows and boxes.

We also evaluated the proposed method using normal quality non-HCP data. DW images of 10 subjects were acquired using a Siemens 3 T TRIO MR scanner following a standard imaging protocol: 30 diffusion directions uniformly distributed on a hemisphere, *b* = 1,000 s/mm^2^, one image with no diffusion weighting, 128 × 128 imaging matrix, voxel size of 2 × 2 × 2 mm^3^, TE = 81 ms, TR = 7,618 ms. As shown in Fig. 12, our method obtains ODFs that are more consistent and exhibit stronger directionality. Visible differences are marked using arrows and boxes. For simple averaging, the ODF glyphs are generally shorter, indicating weaker directionality. In contrast, our method gives sharper and longer ODF glyphs, indicating its superiority.

## Discussion

In this paper, we propose a novel patch-based mean-shift algorithm for constructing diffusion templates. Our method is less sensitive to outliers and is able to deal with inter-subject fiber dispersion. Experimental results confirm that our method yields improvements over the commonly used simple averaging method and generates diffusion templates with cleaner fiber orientations and less artifacts caused by orientation dispersion.

### Reasons for Effectiveness

Most template construction methods have been focused on improving structural alignment in the *x*-space, but less so in the *q*-space. This work presents an effort in the direction of improving microstructural alignment in the *q*-space by using a patch matching mechanism with the mode-seeking mean shift algorithm. The resulting improvements can be attributed mainly to the following factors: (1) Concurrent consideration of both *x*-space and *q*-space allows more fine-grained alignment. We have in fact shown that patch matching in the *x*-*q* space results in good edge preservation^17^; (2) Matching in this joint *x*-*q* space is in general simpler and more reliable because diffusion signal profiles are generally smooth with more predictable shapes; and (3) The mode is more robust to outliers than the mean.

### Future Directions

Future efforts to improve the proposed framework will include (1) Extending the current framework to cater to more general *q*-space sampling methods, including multi-shell and Cartesian sampling schemes. This involves not only considering the directional aspects of the data but also the signal decay with respect to the changes of diffusion weighting. This also involves a more general representation in the *q*-space to take into account both changes in gradient direction and diffusion weighting; see for example Chen *et al*.’s work^17^; (2) Incorporating other methods with possibly better mathematical properties and robustness to outliers and noise than the mean-shift algorithm; (3) Incorporating pre-screening strategies to discard early mismatching patches so that the computational cost can be reduced; (4) Incorporating more sophisticated features for better *q*-space patch matching; see for example Chen *et al*.'s work^27^ and (5) Improving scalability to cope with populations involving hundreds or thousands of subjects. Our current implementation stores all the weights resulting from patch matching and is hence memory demanding.

## Method

### Overview

**Figure 13:**
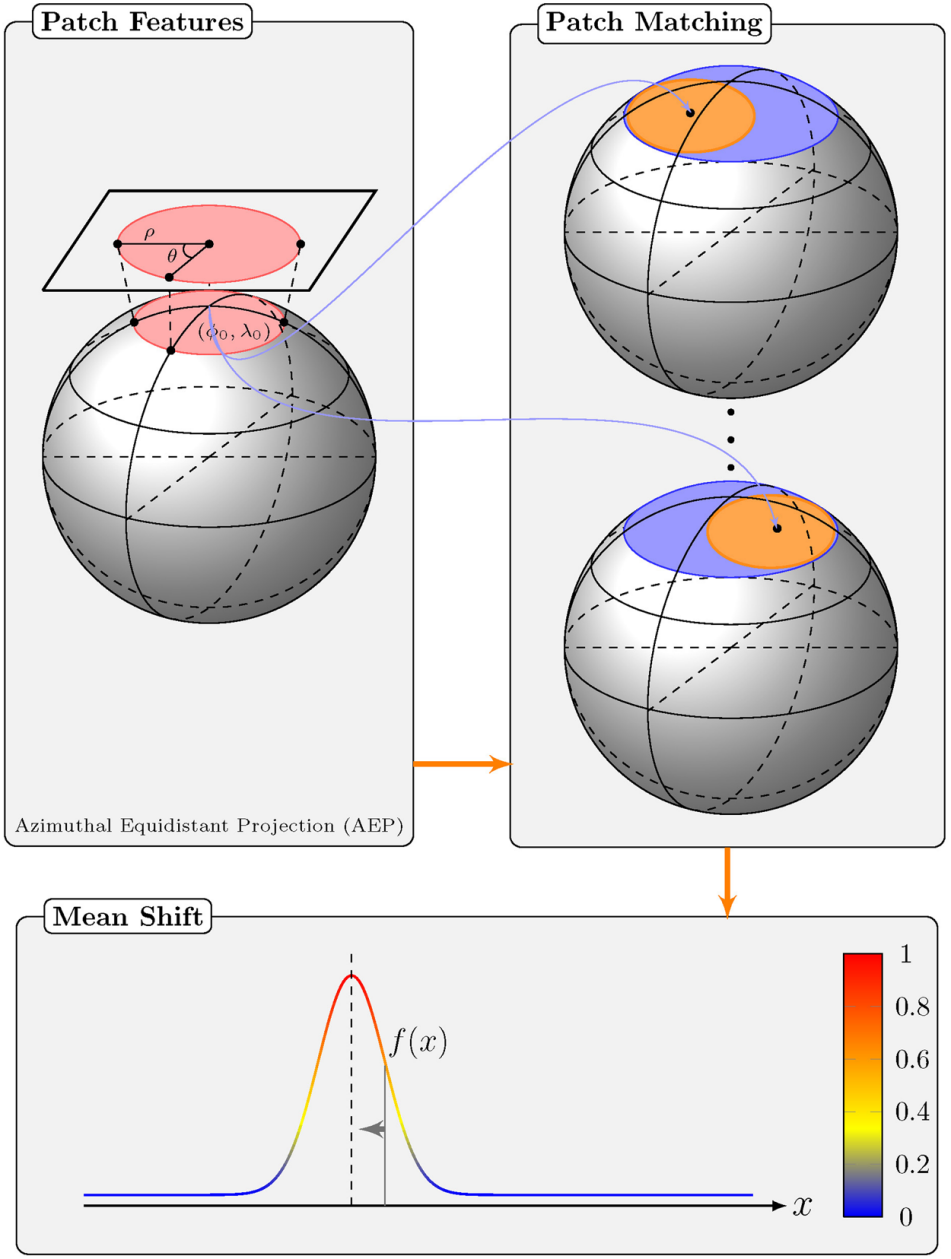
Method overview. Three components of our method: (1) Computation of patch features: The spherical patches are mapped to a disc by using AEP so that rotation invariant patch features can be computed; (2) Patch matching: Using the computed patch features, patch matching is performed in a local *x*-*q* space neighborhood; (3) Mean-shift estimation: The mean shift algorithm is used to seek the most probable signal at each point in *x*-*q*-space.

Our method employs neighborhood matching in *q*-space for effective template construction. For each point in the *x*-*q* space, (**x**_*i*_, **q**_*k*_), where ${{\bf{x}}}_{i}\in {{\mathbb{R}}}^{3}$ is a voxel location and ${{\bf{q}}}_{k}\in {{\mathbb{R}}}^{3}$ is a wavevector, we define a spherical patch, ${{\mathscr{P}}}_{i,k}$, centered at **q**_*k*_ with fixed *q*_*k*_ = |**q**_*k*_| and subject to a neighborhood angle *α*_*p*_. The diffusion signals on this spherical patch are mapped to a disc using azimuthal equidistant projection (AEP) before computing the rotation invariant features via polar complex exponential transform (PCET)^19^ for patch matching. The similarity weights resulting from patch matching will be used in the mean shift algorithm to determine the most probable signal at each point in *x*-*q* space. See Fig. 13 for an overview. Each step is detailed below.

### Patch Features

To make it easier to compute and compare features of a patch on a sphere, we use azimuthal equidistant projection (AEP)^18^ to map the coordinates on a sphere to a flat plane. Azimuthal equidistant projection (AEP) is a one-to-one mapping that preserves the distances and angles between points along the longitudinal lines originating from a reference point. The reference point (*ϕ*_0_, *λ*_0_), with *ϕ* being the latitude and *λ* being the longitude, corresponds in our case to the center of the spherical patch and will be projected to the center of a disc. Viewing the reference point as the ‘North pole’, all points along a given azimuth, *θ*, will project along a straight line from the center of the disc. In the projection plane, this line subtends an angle *θ* with the vertical. The distance from the center to another projected point is given as *ρ*. The relationship between (*ϕ*, *λ*) and (*ρ*, *θ*) is given as^18^

4 $$\begin{array}{rcl}\cos \,\rho  & = & \sin \,{\varphi }_{0}\,\sin \,\varphi +\,\cos \,{\varphi }_{0}\,\cos \,\varphi \,\cos \,(\lambda -{\lambda }_{0}),\\ \tan \,\theta  & = & \frac{\cos \,\varphi \,\sin \,(\lambda -{\lambda }_{0})}{\cos \,{\varphi }_{0}\,\sin \,\varphi -\,\sin \,{\varphi }_{0}\,\cos \,\varphi \,\cos \,(\lambda -{\lambda }_{0})}.\end{array}$$

The projection can be described as **q** → (*q*, *ϕ*, *λ*) → (*q*, *ρ*, *θ*). Note that, since the diffusion signals are antipodal symmetric, we map antipodally all the points on the sphere to the same hemisphere as the reference point prior to performing AEP. After projection, the *q*-space spherical patch ${\mathscr{P}}$ is mapped to a 2D circular patch $\widehat{{\mathscr{P}}}$.

After AEP, we proceed to compute the rotation invariant features. A number of rotation-invariant features have been proposed in the literature, such as the popular Zernike moments (ZMs)^28^, pseudo-Zernike moments (PZMs)^29^, and polar complex exponential transform (PCET)^19^. PCET transforms the signals onto a set of orthogonal basis that is complex-valued. Taking the absolute values of the complex transform coefficients results in a set of features that are not dependent on the orientation of the underlying domain. Compared with ZMs/PZMs, the computation cost of PCETs is extremely low. In addition, the PCETs are numerical more stable especially when the order of the transform is increased. For these reasons, PCET is used in this work. PCET with order *n*, |*n*| = 0, 1, 2, …, ∞, and repetition *l*, |*l*| = 0, 1, 2, …, ∞, of AEP-projected signal profile *S*(**x**, *q*, *ρ*, *θ*) is defined as

5 $${M}_{n,l}(\widehat{{\mathscr{P}}})=\frac{1}{\pi }{\int }_{({\bf{x}},q,\rho ,\theta )\in \widehat{{\mathscr{P}}}}\,{[{H}_{n,l}(\rho ,\theta )]}^{\ast }\,S({\bf{x}},q,\rho ,\theta )\rho \,{\rm{d}}\rho \,{\rm{d}}\theta ,$$

where [·]* denotes the complex conjugate and *H*_*n*,*l*_(*ρ*, *θ*) is the basis function defined as

6 $${H}_{n,l}(\rho ,\theta )={e}^{i2\pi n{\rho }^{2}}\,{e}^{il\theta }.$$

For each patch $\widehat{{\mathscr{P}}}$ consisting of signal vector ${\bf{S}}(\widehat{{\mathscr{P}}})$, the associated PCET features $\{|{M}_{n,l}(\hat{{\mathscr{P}}})|\}$ computed up to maximum order *m* (i.e., −*m* ≤ *l*, *n* ≤ *m*) are concatenated into a feature vector ${\bf{M}}(\widehat{{\mathscr{P}}})$.

### Patch Matching

The similarity of a reference patch ${\widehat{{\mathscr{P}}}}_{i,k}$ with another patch ${\widehat{{\mathscr{P}}}}_{j,l}(d)$ associated with the *d*-th subject is characterized by weight

7 $${w}_{i,k;j,l}(d)=\frac{1}{{Z}_{i,k}}\,\exp \,\{-\frac{{\Vert {\bf{M}}({\widehat{{\mathscr{P}}}}_{i,k})-{\bf{M}}({\widehat{{\mathscr{P}}}}_{j,l}(d))\Vert }_{2}^{2}}{{h}_{{\bf{M}}}^{2}(i,k)}\}\,\exp \,\{-\frac{{\Vert {{\bf{x}}}_{i}-{{\bf{x}}}_{j}\Vert }_{2}^{2}}{{h}_{{\bf{x}}}^{2}}\},$$

where *Z*_*i*,*k*_ is a normalization constant to ensure that the weights sum to one, i.e.,

8 $${Z}_{i,k}=\sum _{d=1}^{D}\,\sum _{({{\bf{x}}}_{j},{{\bf{q}}}_{l})\in {{\mathscr{V}}}_{i,k}}\,\exp \,\{-\frac{{\Vert {\bf{M}}({\widehat{{\mathscr{P}}}}_{i,k})-{\bf{M}}({\widehat{{\mathscr{P}}}}_{j,l}(d))\Vert }_{2}^{2}}{{h}_{{\bf{M}}}^{2}(i,k)}\}\,\exp \,\{-\frac{{\Vert {{\bf{x}}}_{i}-{{\bf{x}}}_{j}\Vert }_{2}^{2}}{{h}_{{\bf{x}}}^{2}}\}.$$

Here *h*_**M**_(*i*, *k*) is a parameter controlling the attenuation of the exponential function. As in Coupé *et al*.’s paper^30^, we set ${h}_{{\bf{M}}}(i,k)=\sqrt{2\beta \,{\hat{\sigma }}_{i,k}^{2}|{\bf{M}}({\widehat{{\mathscr{P}}}}_{i,k})|}$, where *β* is a constant^30^, ${\hat{\sigma }}_{i,k}^{2}$ is the estimated noise standard deviation, which can be computed globally^31^ or spatial-adaptively^30^. The former is used in this paper. Parameter ${h}_{{\bf{x}}}=\sqrt{2}{\sigma }_{{\bf{x}}}$ controls the attenuation of the second exponential function, where *σ*_**x**_ is a scale parameter estimated from the image background. $|{\bf{M}}({\widehat{{\mathscr{P}}}}_{i,k})|$ denotes the length of the vector ${\bf{M}}({\widehat{{\mathscr{P}}}}_{i,k})$.

Given *D* subjects, a “mean” signal can be computed based on the weights resulting from patch matching:

9 $$\bar{S}({{\bf{x}}}_{i},{{\bf{q}}}_{k})=\sqrt{[\sum _{d=1}^{D}\,\sum _{({{\bf{x}}}_{j},{{\bf{q}}}_{l})\in {{\mathscr{V}}}_{i,k}}\,{w}_{i,k;j,l}(d)\,{S}^{2}({{\bf{x}}}_{j},{{\bf{q}}}_{l};d)]-2{\sigma }^{2}},$$

where *S*(**x**_*i*_, **q**_*k*_; *d*) is the measured signal associated with the *d*-th subject at location ${{\bf{x}}}_{i}\in {{\mathbb{R}}}^{3}$ with wavevector ${{\bf{q}}}_{k}\in {{\mathbb{R}}}^{3}$. ${{\mathscr{V}}}_{i,k}$ is a local *x*-*q* space neighborhood associated with (**x**_*i*_, **q**_*k*_), defined by a radius *r*_*s*_ in *x*-space and an angle *α*_*s*_ in *q*-space. Note the bias associated with the Rician noise distribution is removed in this process^31^. *σ* is the Gaussian noise standard deviation that can be estimated from the image background^31^. Without patch matching, a “simple averaging” version of (9) is given as

10 $$\bar{S}({{\bf{x}}}_{i},{{\bf{q}}}_{k})=\sqrt{\frac{1}{D}\,\sum _{d=1}^{D}\,{S}^{2}({{\bf{x}}}_{i},{{\bf{q}}}_{k};d)-2{\sigma }^{2}}\mathrm{.}$$

### Mean Shift

Given a set of diffusion signal profiles $\{S({{\bf{x}}}_{j},{{\bf{q}}}_{l};d):({{\bf{x}}}_{j},{{\bf{q}}}_{l})\in {{\mathscr{V}}}_{i,k},\,d=1,\ldots ,D\}$, we want to determine the modal profile $\tilde{S}({{\bf{x}}}_{i},{{\bf{q}}}_{k})$. This is achieved using a mean shift algorithm^15^ that is modified to take advantage of the patch matching mechanism described above. Mean shift is a non-parametric algorithm for locating the maxima of a density function and is hence a mode-seeking algorithm. It is an iterative algorithm where the mean is progressively updated by using the mean computed in the previous iteration as the reference for computing sample similarity. Let a kernel function *K*(*x*_*i*_ − *x*) be given. This function determines the weight of nearby points for re-estimation of the mean. Typically a Gaussian kernel on the distance to the current estimation is used, $K({x}_{i}-x)={e}^{-c{\Vert {x}_{i}-x\Vert }^{2}}$. The weighted mean of the density in the window determined by *K*:

11 $$m(x)=\frac{{\sum }_{{x}_{i}\in N(x)}K({x}_{i}-x){x}_{i}}{{\sum }_{{x}_{i}\in N(x)}K({x}_{i}-x)},$$

where *N*(*x*) is the neighborhood of *x*, a set of points for which *K*(*x*) ≠ 0. The difference *m*(*x*) − *x* is called *mean shift*. The mean-shift algorithm now sets *x* ← *m*(*x*), and repeats the estimation until *m*(*x*) converges.

We first note that the weights computed using (7) is dependent on the signal vector ${\bf{S}}(\widehat{{\mathscr{P}}})$ of a patch $\widehat{{\mathscr{P}}}$. To explicitly express this dependency, we write ${w}_{i,k;j,l}(d):=w\,(\bar{{\bf{S}}}({\hat{{\mathscr{P}}}}_{i,k}),\,{\bf{S}}({\hat{{\mathscr{P}}}}_{j,l}(d)))$. Note that we have made here the mean signal vector $\bar{{\bf{S}}}({\widehat{{\mathscr{P}}}}_{i,k})$ the reference for weight computation. Our implementation of the mean shift algorithm involves the following steps. For iteration *t* = 1, 2, …, *T*,

1. Update weights ${w}_{i,k;j,l}^{(t)}(d)=w\,({\bar{{\bf{S}}}}^{(t-\mathrm{1)}}({\hat{{\mathscr{P}}}}_{i,k}),\,{\bf{S}}({\hat{{\mathscr{P}}}}_{j,l}(d)))$ based on (7).
2. Update the mean at each location (**x**_*i*_, **q**_*i*_) using (9) with weights $\{{w}_{i,k;j,l}^{(t)}(d)\}$ and {*S*(**x**_*j*_, **q**_*l*_; *d*)} for $({{\bf{x}}}_{j},{{\bf{q}}}_{l})\in {{\mathscr{V}}}_{i,k}$.
3. Repeat steps above with *t* ← *t* + 1.

### Parameter Settings

For all experiments, we use the following parameters:

1. Coupé *et al*.^30^ suggested to set *r*_*s*_ = 2 voxels and *β* = 1, we followed the former, but for the latter we set *β* = 0.1 since we have a greater number of patch candidates by considering the joint *x*-*q* space. Based on the theory of kernel regression, reducing the bandwidth when the sample size is large reduces bias. Results shown in Fig. 14 indicate that a suitable value for *β* is 0.1.

**Figure 14:**
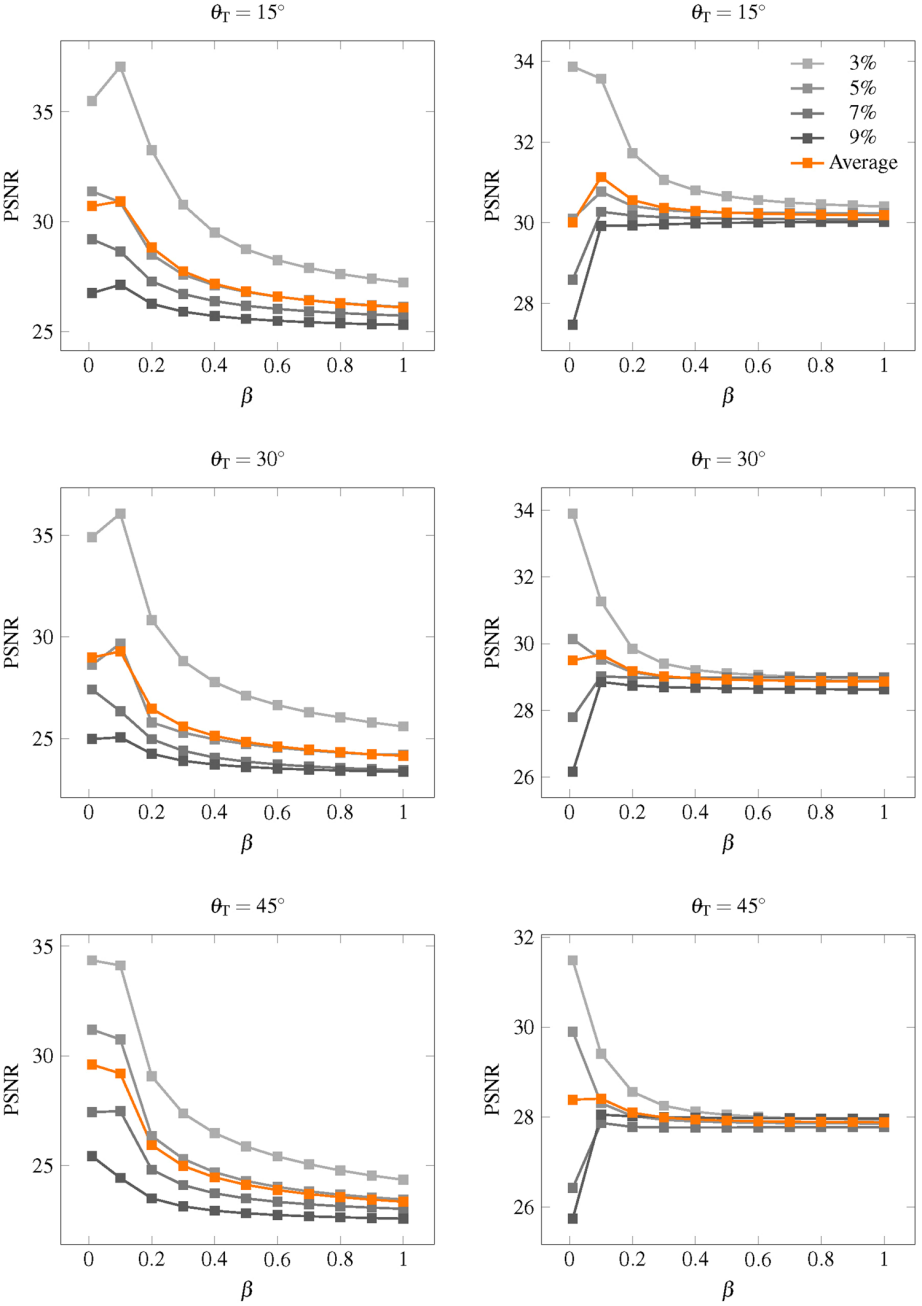
PSNR in relation to *β*. PSNR in relationship to parameter *β* for synthetic dataset with (left column) one direction and (right column) two directions.
2. In our case, the minimal angular separation of the gradient directions is around 15° for each shell. We set the *q*-space neighborhood angle and search angle to twice of this value, i.e., *α*_*p*_ = *α*_*s*_ = 2 × 15° = 30°.
3. We set maximum order *m* = 4 with the consideration of both the quality of rotation-invariant features and the computational efficiency.
4. We compute the mean absolute difference ${tol}$ between the outcomes of two consecutive iterations and stop iterating when ${tol} < \gamma \sigma$, where *σ* is the standard derivation of Gaussian noise and *γ* = 0.001.
